# Advantages and Disadvantages of Educational Email Alerts for Family Physicians: Viewpoint

**DOI:** 10.2196/jmir.3773

**Published:** 2015-02-27

**Authors:** Hani Badran, Pierre Pluye, Roland Grad

**Affiliations:** ^1^Information Technology Primary Healthcare Research GroupDepartment of Family MedicineMcGill UniversityMontreal, QCCanada

**Keywords:** theory of planned behavior, continuing medical education, educational email alerts, electronic knowledge resources, family physicians, health informatics, knowledge translation, primary health care

## Abstract

**Background:**

Electronic knowledge resources constitute an important channel for accredited Continuing Medical Education (CME) activities. However, email usage for educational purposes is controversial. On the one hand, family physicians become aware of new information, confirm what they already know, and obtain reassurance by reading educational email alerts. Email alerts can also encourage physicians to search Web-based resources. On the other hand, technical difficulties and privacy issues are common obstacles.

**Objective:**

The purpose of this discussion paper, informed by a literature review and a small qualitative study, was to understand family physicians’ knowledge, attitudes, and behavior in regard to email in general and educational emails in particular, and to explore the advantages and disadvantages of educational email alerts. In addition, we documented participants’ suggestions to improve email alert services for CME.

**Methods:**

We conducted a qualitative descriptive study using the “Knowledge, Attitude, Behavior” model. We conducted semi-structured face-to-face interviews with 15 family physicians. We analyzed the collected data using inductive-deductive thematic qualitative data analysis.

**Results:**

All 15 participants scanned and prioritized their email, and 13 of them checked their email daily. Participants mentioned (1) advantages of educational email alerts such as saving time, convenience and valid information, and (2) disadvantages such as an overwhelming number of emails and irrelevance. They offered suggestions to improve educational email.

**Conclusions:**

The advantages of email alerts seem to compensate for their disadvantages. Suggestions proposed by family physicians can help to improve educational email alerts.

## Introduction

### Background

Educational email alerts have the potential to improve the quality of health care services, but present some disadvantages. Email alerts have been associated with learning, practice change, and expected benefits for patient health, among other outcomes [1,2]. They can be used for two-way knowledge translation, a process that involves sending evidence-based clinical recommendations to physicians and then receiving their constructive feedback [3,4].

Two examples of educational email alerts are “Daily-POEMs” (Patient-Oriented Evidence that Matters) and “Highlights from e-Therapeutics+” [1,3,5]. These alerts support continuing education programs that involve more than 10,000 Canadian physicians and pharmacists [3]. First, Daily-POEMs are tailored for family physicians. POEMs are synopses of original research and systematic reviews, selected after scanning and critically appraising new articles from more than 100 journals. Second, Highlights are treatment recommendations tailored for a primary care audience. They are based on a synthesis of research results and systematic reviews, and graded using the Strength of Recommendation Taxonomy System. They consist of key updated paragraphs from a Web-based text called e-Therapeutics+, published by the Canadian Pharmacists Association (CPhA). Highlights have been delivered to members of the College of Family Physicians of Canada since 2010 and to CPhA members since 2012.

However, physicians face email-related obstacles such as technical difficulties [3]. Privacy and security of email communication remain of major concern [6]. Other issues are changes of email address or service provider [7]. In addition, physicians complain about “email fatigue”, that is, too many emails and lack of time [8].

Understanding physicians’ knowledge, attitude, and behavior regarding email alerts can help to address obstacles associated with email as an educational channel. We found studies on the use of information derived from educational emails by physicians, but no studies on the advantages or disadvantages of email for the delivery of educational material. Therefore, our main objective was to explore the viewpoint of family physicians on advantages and disadvantages of educational email alerts.

By 2009, 80.3% of Canadians were using the Internet on a regular basis; the main reason for Internet usage was email, with 93.0% of Canadians using it for email [9]. Almost all physicians use email as do 94.7% of Canadians with a university degree [9]. In the clinical setting, physicians use email to consult with colleagues, obtain laboratory data, follow up with staff about patient care issues, and learn about new research findings [3,10]. When used as a method of communication between physician and patient, email can improve the doctor patient relationship, despite privacy and security concerns [6,11]. According to the 2010 Canadian National Physician Survey, 50.1% of family physicians email their colleagues for clinical purposes [12]. Increasingly, family physicians are using email for their continuing education [13]. However, in a review of the literature guided by a specialized librarian, we found no studies on family physicians’ perception about positive and negative aspects of educational emails. This justifies our exploratory work on the family physician viewpoint.

### Educational Email Alerts

We identified nine studies on physicians’ use of information from educational email alerts. Three studies globally evaluated satisfaction and usefulness of receiving health information via email [14-16]. In these studies, users of email alerts reported high levels of satisfaction and perceived them to be useful for continuing education. A fourth study evaluated the effect of email alerts on information awareness and knowledge acquisition [17]. While subscribers of email alerts became more familiar with the recent literature, their medical knowledge was not improved. A fifth study evaluated the effect of email alerts on subsequent information retrieval by physicians and demonstrated that users of email alerts are more likely to search for information [18]. The sixth study examined self-reported cognitive impact of emailed synopses of recently published clinical research, and indicated that email alerts have a positive impact [19]. Subsequently, another study indicated that email alerts are infrequently retrieved after initial reading [20]. Finally, two studies suggested that email dissemination of synopses of systematic literature reviews [21], and of treatment recommendations is associated with anticipated benefits for patient health [3].

### Advantages and Disadvantages of Educational Email Alerts

While we found no studies that specifically focused on advantages and disadvantages of emails according to health care professionals, four studies and one literature review on email [2,7,8,11,22] did mention this in passing (Table 1). For example, physicians are knowledgeable and familiar with email as a way of educating and communicating with medical students, by sending evidence-based clinical recommendations and individualized feedback [2]. In contrast, physicians can face technical difficulties when using email, because of slow Internet connections or software incompatibility. They complain about receiving too much information by email while not having time to read it [8]. Therefore, our research questions were: what are the advantages and disadvantages of educational email alerts from the physicians’ viewpoint?

**Table 1:** Advantages and disadvantages of email, as mentioned in the literature.

First author and date of study	Design, participants, setting, intervention, data collection and analysis	Types of advantage	Types of disadvantage
Barnhart 2010	Design: Cross-sectional Participants: 69 medical students Setting: Illinois University, Department of Family and Community Medicine Intervention: Educational emails containing clinical questions from standardized patients Data collection: Email replies Data analysis: Descriptive statistical	Good for training purposes. Possibility to receive feedback to improve the content. Easy to manage, as medical students are familiar with email.	Emails did not cover all educational topics.
Bennett 2005	Design: Survey Participants: 2200 community-based physicians Intervention: Survey Setting: United States Data collection: Survey responses Data analysis: Descriptive statistical	The Internet is an important tool for practice. Hand-held computers are useful educational tools, especially for drug information.	Information overload. Specific information often not found. Socio-technical difficulties eg, navigation, and searching. Internet connection sometimes too slow.
Kenny 2000	Design: Literature review of the usage of telecommunication (including email) Participants: Primary research studies involving general practitioners	Can be used anytime. Enhances the relationship between the doctor and the patient.	Family physicians fear being overwhelmed by patient inquires by email. The medical defense union has concerns about the security of email.
Moyer 2002	Design: Cross-sectional survey Participants: 476 outpatients, 126 family physicians, and 16 clinical staff. Setting: United States Intervention: Survey Data collection: Survey responses Data analysis: Descriptive statistical	Improves the relationship with patients. A good way to follow up with patients. A fast way to communicate with colleagues for consultation and lab results.	Email from patients would add to work load and not substitute for other tasks. Fear of being overwhelmed by patient email. Security concerns. Costs for implementation, integration, and maintenance of new systems.
Seguin 2004	Design: Randomized controlled trial Setting: Ontario, Canada Participants: 2397 family physicians Intervention: Survey Data collection: Survey responses Data analysis: Descriptive statistical	All physicians with academic practices had email addresses. Rapid method to obtain survey data. Email encourages physicians to write more than they would by regular mail.	Email addresses are subject to rapid change. Email messages are too easy to delete. Joint (family or business email) accounts reduce the chance of checking email.

## Methods

### Study Design

A qualitative descriptive study [23] was conducted through semi-structured face-to-face interviews with 15 family physicians. Participants were members of the Department of Family Medicine, McGill University, who had received an email to briefly explain the study. An invitation to participate in the study was emailed to 290 family physicians affiliated with this department. Of 17 family physicians who replied, two were not interviewed because we could not arrange an interview. Yet, the saturation of data was confirmed during the interviews, through the repetition of similar answers to our interview questions. We decided to conduct semi-structured face-to-face individual interviews because we were interested mainly in their individual experience and perceptions. The interview was conducted in four main parts (see Multimedia Appendix 1).

### Part 1. Demographic Questions

In this part, four demographic questions were asked (ie, age, years of practice, practice setting (s), and special interests).

### Part 2. Participants’ Knowledge, Attitude, and Behavior (Theory of Planned Behavior) Regarding Email

In this part, four questions were asked to assess participants’ experience (knowledge, attitude, and behavior) with email in general and educational email in particular. Using the Theory of Planned Behavior [24], we explored the daily experience of participants with email (knowledge), their psychological reaction toward email (attitude), and their behavior when they received an email. This theory was chosen because it is validated and commonly used for assessing health education programs and health care professional behavior [25]. The interview questions included: *Knowledge:* Please describe your daily experience with email, *Attitude:* How do you usually feel about email, eg, welcoming, disliking, feeling overwhelmed, or something else?, and *Behavior:* What do you usually do when you receive email, eg, reading, deleting, flagging, ignoring, saving, classifying, or anything else?

### Part 3. Perception of the Advantages and Disadvantages of Educational Email

In this part, participants were asked three questions about their preferences for Continuing Medical Education (CME), specifically, the advantages and disadvantages of educational email.

### Part 4. Recommendations to Improve Educational Email

In this part, participants were asked about their recommendations to improve educational email alerts.

### Data Analysis

Interviews were transcribed, reviewed, summarized, and then a deductive-inductive thematic analysis was conducted [26]. To this end, we assigned preliminary themes based on the Theory of Planned Behavior, the literature review, and the interview guide, and then searched for emerging themes. The inductive process involved the identification of themes through careful reading and re-reading of the data in six sessions. The coding process was conducted in six stages [26]: (1) we developed the code manual, (2) we tested the reliability of the codes, (3) we summarized the data and identified the initial themes, (4) we applied a template, (5) we connected the codes in accordance with the process of discovering themes and patterns in the data, and (6) we corroborated and legitimated coded themes, especially the item-related codes.

Finally, the results were reviewed by two of us (PP, RG). We prepared a table of findings for each group of questions related to: (1) demographic data, (2) participants’ preference for continuing education activities, (3) participants’ experience with email, (4) participants’ perception of the advantages and disadvantages of educational email, and (5) participants’ recommendations to improve educational email. The data analysis process and final results were discussed with colleagues who conduct research in the fields of Information Technology and Primary Health Care. We distributed a report of the data analysis process and our results to members of the Information Technology Primary Care Research Group, and we allowed a week for detailed reading and commenting. Then, at one meeting, group members helped to interpret the results.

### Ethical Approval

This study was conducted according to the ethical principles stated in the declaration of Helsinki. Ethical approval was obtained from the McGill University Institutional Review Board (IRB).

## Results

### Part 1. Demographic Results

A total of 15 family physicians were interviewed (nine male and six female). Nine family physicians were working in academic health science centers, university, or teaching units while the other six worked in community-based clinics. The participants’ number of years in practice ranged from 9 to 38. Five participants indicated no clinical focus to their practice, while 10 expressed a special focus such as maternity and newborn care (Table 2).

Participants were involved in many CME activities (eg, conferences and Web-based activities) (see Table 3). While six family physicians mentioned no specific preference for CME activities, five family physicians expressed interest in Web-based activities (eg, educational email), three expressed interest in group learning (eg, conferences and clinical rounds), and one family physician expressed interest in reading magazines and journals.

All interviews were done face-to-face in participants’ offices. Interviewees were welcoming and co-operative: 11 of 15 gave adequate time for the interview while only four seemed rushed. All interviewees answered all questions. Based on our interpretation of viewpoints, results are presented in three parts. First, participants reported their knowledge, attitude, and behavior regarding email in general, and educational email in particular. Second, they specifically reported advantages and disadvantages of educational email. Third, they proposed recommendations to improve educational email.

**Table 2:** Participants’ demographic data.

Participant	Years of practice	Special focus	Work setting
P1	38	No	AHSC^a^(university affiliated teaching hospital)
P2	37	No	AHSC (university)
P3	36	No	Private office
P4	35	Global health; health care of the elderly; mental health	University affiliated teaching hospital
P5	34	Health care of the elderly; home care	AHSC
P6	32	No	Private office
P7	31	No	AHSC
P8	30+	Adult ADHD	Private office
P9	23	Child and adolescent health care	Private office
P10	20	Maternity and newborn care	AHSC (university)
P11	20	Maternity and newborn care; immigrant and refugee care	Community clinic; AHSC (Family medicine teaching unit)
P12	12	Hospital medicine	Private office
P13	9	Health care of the elderly; hospital medicine; diabetic foot and wound clinic	AHSC (university); Nursing home
P14	9	Care of patients with sexually transmitted disease	Private office
P15	7	Maternity and newborn care; tropical and travel medicine	AHSC

^a^AHSC: academic health science center

**Table 3:** Continuing medical education (CME) activities reported by the participants (n=15).

Type of CME activities	n (%)
Group learning (eg, conferences)	13 (87%)
Online learning (eg, email alerts)	11 (73%)
Self-learning (eg, reading journals)	9 (60%)
Teaching or research	9 (60%)
Journal club / lunch time meetings	4 (27%)
University courses	3 (20%)
Clinical rounds	3 (20%)

### Part 2. Participants’ Knowledge, Attitude, and Behavior Regarding Email

#### Knowledge

Of 15 family physicians, 13 said they were familiar with email and checked their email from one to four times per day. These regular users checked email for clinical, educational, and personal reasons. Regular users received from 10 to 100 emails per day. In contrast, the two family physicians who were not familiar with email, used email for personal communication and checked it two or three times a week. Their two main reasons for not being regular users were (1) limited time because of family obligations, and (2) issues with technology (such as familiarity).

#### Attitude

Of 15 family physicians, nine family physicians felt comfortable with and liked email, three expressed a neutral attitude, and three disliked or felt overwhelmed by email. Only three family physicians were not overwhelmed by the volume of email, and only one family physician expressed a concern regarding confidentiality when using email in communication with patients.

#### Behavior

The 15 family physicians mentioned they scanned emails by reading the title, and prioritized them according to urgency and relevance. First, they replied to the urgent emails, then, time permitting, they replied to others. Second, they deleted irrelevant email. In addition, 13 family physicians mentioned that they archive important email in a folder, while the other two delete all email after reading. Regarding participants’ behavior toward educational email, all 15 family physicians mentioned they follow the same procedure, namely scan and prioritize.

### Part 3. Perceptions of the Advantages and Disadvantages of Educational Email

#### Advantages

Participants mentioned six types of advantages (see Table 4).

#### Disadvantages

Of 15 family physicians, 12 mentioned disadvantages that we grouped into six main types (see Table 5).

**Table 4:** Advantages of educational email as reported by the participants (n=15).

Advantages	n (%)
Convenient: they are brief and can be “read 24/7”	11 (73%)
Contain valid information family physicians can trust	5 (33%)
Give family physicians the option to use the information	4 (27%)
Constitute an easy way to disseminate information	2 (13%)
Broaden family physician knowledge, eg, raise their awareness	2 (13%)
Regularly received at a specific time	2 (13%)

**Table 5:** Disadvantages of educational emails as reported by the participants (n=15).

Disadvantages	n (%)
Overwhelming, eg, email difficult to manage	6 (40%)
Not relevant to specialized practice	2 (13%)
Time consuming	1 (7%)
Email may cost to use and to maintain	1 (7%)
Educational email is sometimes confused with commercial email (spam)	1 (7%)
Email readability is affected when writers are not professional editors	1 (7%)

### Part 4. Recommendations to Improve Educational Email

Participants provided 23 recommendations, presented in Textbox 1. They suggested five general recommendations such as “Avoid sponsorship by pharmaceutical companies”. They provided six recommendations regarding the informational content of email, for example, “Add a description of the writers’ affiliation”. There were 11 recommendations concerning the design of educational email, such as “Add a link to a discussion board on the topic”.

**Participants’ recommendations to improve educational email.**

General recommendations:

1. Avoid pharmaceutical sponsorship.
2. Clarify the subscription procedure.
3. Maintain the continuity and regularity of the emails.
4. Reduce the price.
5. Send email at a specific time of the day.

Recommendations related to the content:

1. Add a description of the writers’ affiliation.
2. Briefly describe the pathophysiology of the condition.
3. Concentrate on local health and system issues.
4. Email only clinically relevant content.
5. Email only validated content from high quality primary research or knowledge syntheses.
6. Email only up to date content.
7. Provide a summary and a link to the original article(s).

Recommendations related to the email design:

1. Adapt educational email for older readers (eg, larger font).
2. Add a link to a discussion board on the topic.
3. Add a link to archived topics from previous email.
4. Add a printable one page summary.
5. Add a way for readers to ask questions or send inquiries.
6. Avoid complex graphics and provide very simple text.
7. Avoid highly specialized technical functions associated with email.
8. Distinguish the appearance of educational from commercial email.
9. Provide the conclusion and summary in separate sections.
10. Include all information content in the email.
11. Send a reminder email with peer feedback (after few months).

## Discussion

### Principal Findings

No previous studies have specifically focused on the pros and cons of email from a physicians’ viewpoint, although four studies and one review [2,7,8,11,22] have mentioned this in passing (Table 1). This literature suggests physicians are familiar with email for their education and for communication [2]. In addition, it shows that some physicians face technical difficulties when using email, and complain about receiving too much information by email while not having time to read it [8]. Our results are aligned with the literature in that most of our participants were familiar with email, while many felt comfortable and liked using email in their professional life, and some felt overwhelmed by the volume of email they receive.

In addition, our results suggest types of advantages and disadvantages of educational email that were not previously mentioned in the literature. First, with respect to the advantages: (1) educational email can contain valid and trustworthy information, (2) is an easy way to disseminate information to multiple recipients, (3) broadens the spectrum of family physician knowledge, (4) is regularly sent at a specific time, (5) contains brief clinical synopses, and (6) gives the reader an option to use them.

Second, with respect to the disadvantages of educational email not previously mentioned in the literature: (1) educational emails are overwhelming in number and because of the information they contain, (2) they are not relevant to specialized practice, (3) they can resemble commercial email, and (4) some writers of educational email are not professionals.

However, there were two contradictions in the viewpoints expressed. First, physicians do not want advertisements within educational email, in line with Canadian CME policies [27], while they want this service for free. Second, they want brief “bottom-line” information, while asking for more information about the underlying “black-box” process surrounding the submission, peer-review, and editing of research articles.

Similar to usual qualitative research, our exploratory study faces two main limitations: small sample size and researchers’ interpretation. As researchers, we are involved in the evaluation of email-based CME programs [3,4,28]. In addition, the homogeneity of participants and investigators may have limited the scope of participants’ comments. This experience influenced our interpretation of participant viewpoints. In line with the “blind-spot” effect proposed by the anthropologist George Devereux [29], this might have lead us to miss some issues reported by participants. Participants were recruited by email invitation. We interviewed them in their offices. They were very welcoming, interested in our research topic, and 11 of 15 gave plenty of time to the interview. Having said this, in-depth face-to-face interviews with 15 family physicians provided rich data, as participants had a wide range of familiarity with email. In addition, we obtained redundant answers from participants suggesting data saturation was reached.

Finally, all participants made suggestions for improving educational email such as enabling links to a discussion board. A number of their suggestions are relevant to the providers of educational email alerts, namely to use valid studies, to add background information on pathophysiology, to enable a printable summary, and to provide comment boxes.

###  Conclusion

Given email still has some disadvantages as an educational channel, there is room to improve educational email alerts. Hence, information providers would be well advised to consider both the advantages and disadvantages of educational email as suggested by physicians.

## Multimedia Appendix 1

The interview guide.

## Abbreviations

CME: Continuing Medical Education
CPhA: Canadian Pharmacists Association
IAM: Information Assessment Method
POEM: Patient-Oriented Evidence that Matters
