# Supplementary material for: Advantages and Disadvantages of Educational Email Alerts for Family Physicians: Viewpoint
Source: J Med Internet Res. 2015 Feb 27;17(2):e49. doi: 10.2196/jmir.3773 (PMC4376149; doi:10.2196/jmir.3773)
Supplement: Supplementary file 1 [file jmir_v17i2e49_app1.pdf]

Appendix (1): The Interview Guide

INTERVIEW GUIDE

Introduction

To begin, I would like to explain briefly the context of our interview. To our knowledge, no studies have assessed the advantages and disadvantages of educational emails. In our in our interview, I will explore your experience with Continues Medical education activities, preferences, perception of advantages and disadvantages of educational emails and your recommendations to improve the educational services via emails.

The interview will be conducted in two steps (1) we will ask some demographic questions, (2) we will discuss your personal experience and preferences regarding CME activities, (3) we will explore the advantages and disadvantages of educational email alerts, and (4) we will document your recommendations to improve the educational service via email. The interview will last between 20 to 30 minutes depending on our interaction and time available.

- Before we start, do you have any questions?

Demographic questions:

In this part, the interviewees will be asked demographic questions regarding the kind of their practice and years in this practice.

In the beginning, I would like to ask you some demographic questions.

Q1- Would you like to describe yourself as a:

1- Family physician

2- Other speciality physician?

Q2- How long have you been working in your speciality?

Q3- Is your practice focused in any of the following areas?

1- No
2- Addiction medicine
3- Chronic non-cancer pain
4- Developmental disabilities
5- Child and adolescent health
6- Emergency medicine
7- Family practice anesthesia
8- Global health
9- Health care of the elderly
10- Hospital medicine
11- Maternity and newborn care
12- Mental health
13- Occupational medicine
14- Palliative care
15- Prison health
16- Respiratory medicine
17- Sport and exercise medicine
18- Other, please specify?

Q4- What best describes your work settings? Check all that apply.

1- Private office/clinic (excluding free standing walk-in-clinics)
2- Community clinic/community health centre

3- Free standing walk-in-clinic

4- Academic health sciences centre (AHSC)

5- Non-AHSC teaching hospital

6- Community hospital

7- Other hospital

8- Emergency department (in community hospital or AHSC)

9- Nursing home/long term care facility/seniors' residence

10- University

11- Research unit

12- Free-standing lab/diagnostic clinic

13- Administration office/corporate office

14- Other, please specify?

In this part of our interview we will explore your knowledge, attitude and behaviour regarding emails in general and educational email in particular:

First, I would like to ask you some baseline questions about emails and educational email alerts**.**

Q1- Please, describe your daily experience with emails? Probes: Is there a specific reason for that? Can you give me an example? Does this routine experience influence your utilization of InfoPOEMs or Highlights?

Q2- What do you usually feel (good or bad) about emails, e.g., welcoming, disliking, feeling overwhelmed or unsecured, or anything else? Probes: Is there a specific

reason for that? Can you give me an example? Does this usual attitude influence your utilization of InfoPOEMs or Highlights?

Q3- What do you usually do when you receive emails, e.g., reading, deleting, flagging, ignoring, saving, classifying, or anything else? Is there a specific reason? Could you give me an example of that?

Q4- Does this usual behaviour influence your utilization of InfoPOEMs or Highlights?

In this part of our interview, we will discuss your personal experience and preferences regarding the CME activities and the advantages and disadvantages of educational email alerts:

Second, I would like to ask you three general questions regarding your CME experience and preference:

Q1- Can you tell me about your CME activities?

Q2- What kind of CME activities do you prefer?

Q3- What are the advantages and disadvantages of educational email alerts compared to other CME activities?

CONCLUDING QUESTION

Based on your experience with educational email alerts, do you have recommendations to improve the service in general?

PROBE: could you give me an example for each recommendation?

Conclusion:

Finally, I thank you very much and I would ask you if you have any comment about our study, the data collection process or the interview.
